# Maternal exposure to diethylstilbestrol during pregnancy and increased breast cancer risk in daughters

**DOI:** 10.1186/bcr3649

**Published:** 2014-04-30

**Authors:** Leena Hilakivi-Clarke

**Affiliations:** 1 Department of Oncology, Georgetown University Medical Center, Research Building, Room E407, 3970 Reservoir Road, NW, Washington, DC 20057, USA

## Abstract

The idea that susceptibility to breast cancer is determined not only through inherited germline mutations but also by epigenetic changes induced by alterations in hormonal environment during fetal development is gaining increasing support. Using findings obtained in human and animal studies, this review addresses the mechanisms that may explain why daughters of mothers who took synthetic estrogen diethylstilbestrol (DES) during pregnancy have two times higher breast cancer risk than women who were not exposed to it. The mechanisms likely involve epigenetic alterations, such as increased DNA methylation and modifications in histones and microRNA expression. Further, these alterations may target genes that regulate stem cells and prevent differentiation of their daughter cells. Recent findings in a preclinical model suggest that not only are women exposed to DES *in utero* at an increased risk of developing breast cancer, but this risk may extend to their daughters and granddaughters as well. It is critical, therefore, to determine if the increased risk is driven by epigenetic alterations in genes that increase susceptibility to breast cancer and if these alterations are reversible.

## 

In the early 1990s, Drs Barker [1] and Trichopoulos [2] proposed that the risk of developing cardiovascular diseases and breast cancer, respectively, might be programmed during fetal development. According to Dr Barker, inadequate nutrition during early life and consequent low birth weight may program some cells in the fetus to have metabolic characteristics that can lead to increased cardiovascular disease risk later in life [3]. Dr Trichopoulos and collaborators discovered that high birth weight and other indicators of exposure to high hormone levels *in utero* increase later breast cancer risk [2]. Both hypotheses have been confirmed in numerous studies, and it also has been shown that the effects are independent of adult body weight.

The reason why high birth weight is linked to increased breast cancer risk may be due to elevated pregnancy estrogenic environment [4], but also to changes in leptin, adiponectin, glucose, insulin and insulin-like growth factor levels. The major difficulty in determining, for example, if high *in utero* estrogen levels increase later breast cancer risk is that data must be available for both fetal hormonal environment and breast cancer incidence approximately 50 years later. These data can be obtained from daughters whose mothers took the synthetic estrogen diethylstilbestrol (DES) during pregnancy.

In the early 1940s, physicians started prescribing DES to pregnant women who exhibited signs of being in danger of having a miscarriage. The rationale for this practice was that miscarriage is preceded by a drop in estrogen levels, and providing women with estrogen might help to sustain the pregnancy. However, Herbst and colleagues [5] discovered in the early 1970s that DES caused rare reproductive system cancers in young daughters; therefore, cohorts were established to follow the offspring of the exposed women. At the present time five centers in the US are still recruiting women and men who were exposed to DES *in utero* for Continuation of Follow-up of DES-Exposed Cohorts trial (ClinicalTrials.gov ID NCT00340600).

In this review, findings related to *in utero* DES exposure and breast cancer are discussed for the purpose of weighing evidence as to whether fetal hormonal environment can impact breast cancer risk in women several decades later. Since causal studies can readily be performed using animal models, findings obtained in DES-exposed mouse and rat offspring also are discussed. Importantly, animal studies were done prior to any epidemiological studies addressing a possible link between maternal DES exposure and breast cancer risk among daughters could be performed. By the 1980s, exposed daughters in the cohorts began to be old enough to develop breast cancer and several human studies have been performed since to determine if maternal exposure to DES during pregnancy increases an offspring’s breast cancer risk.

## Maternal exposure to diethylstilbestrol during pregnancy and breast cancer risk among daughters

The idea that DES prevents miscarriage was tested in the 1940s by Smith and Smith [6,7] in a clinical study and they reported fewer spontaneous abortions, preterm labor, and pre-eclampsia in women receiving DES during pregnancy. However, since the controls were not selected randomly and the study was not performed blindly, both the study design and interpretation of the data were compromised. It later became apparent that DES treatment was ineffective at preventing miscarriage [8] and now it is known that it in fact increases the risk of miscarriage [9].

The exact number of women who used DES is not known, but it is estimated to have been between 5 to 10 million women worldwide. These numbers may be an underestimation, as DES was marketed by several drug companies and under several different trade names and was included in some prenatal vitamin preparations. Further, in 1954 DES use was approved for livestock to promote growth, and two years later approximately two-thirds of US beef cattle were treated with DES [10]. The US Food and Drug Administration banned DES in 1971 for pregnant women and in 1973 for cattle when physicians reported several cases of clear cell adenocarcinoma of the vagina and cervix in young women whose mothers took DES [5]. However, DES use continued in Europe and other parts of the world after 1971. For example, according to *DES Timeline *[11], DES prescriptions continued until 1977 in France, 1980 in Spain, 1981 in Italy and 1983 in Hungary.

### Findings in animal studies

Since estrogens are linked to increased breast cancer risk, and since maternal DES exposure induces cancers in the offspring’s reproductive tissues, a concern rose that daughters exposed to DES might exhibit increased risk of developing breast cancer. Animal studies were first done to investigate the effect of *in utero* or neonatal DES exposure on mammary gland development [12-15] and later mammary tumorigenesis [16-21]; they are summarized in Table 1. The amount of DES given to pregnant mice or rat dams varied from study to study (0.2 to 12,000 μg/day, which translates in rats to approximately 1 μg/kg to 60 mg/kg DES per day; in pregnant women the daily DES dose ranged from 100 μg/kg to 2 mg/kg), as did the route of administration (subcutaneous injection or via feed) and mammary tumor model used (spontaneous, carcinogen-induced, or ACI rats, which develop mammary tumors upon estrogen exposure).

**Table 1:** **Summary of results on mammary gland development and mammary tumorigenesis obtained in mice and rats exposed to diethylstilbestrol *in utero*, at birth, during neonatal period (between days 0 and 5) or postnatally**

**Reference**	**Model**	**DES dose** **(total)**	**Time of administration**	**Effect on reproductive system**^**a**^	**Effect on mammary gland or mammary cancer**
**Effects on mammary gland**					
Nagasawa *et al.* 1978 [12]	Balb/cfC3H mice	5 μg	0-5 Postnatal	100% no CL	Hyperplasia↑
		20 μg	0-5 Postnatal	100% no CL	
Boylan 1978 [13]	Rat	1.2 μg	Week 2 G	Percentage live deliveries: 97/F^1^ 94%	Normal
		12 μg	Week 2 G	50%	Normal
		60 μg	Week 2 G	27%	Normal
		120 μg	Week 2 G	33%	Normal
		1,200 μg	Week 2 G	No surviving pups	
		12,000 μg	Week 2 G	No surviving pups	Normal
		1.2 μg	Week 3 G	81/F^1^ 78%	Slightly enlarged nipples
		120 μg	Week 3 G	62/F^1^ 57%	
		12,000 μg	Week 3 G	No surviving pups	
Bern *et al.* 1987 [14]	Balb/c mice	5 × 10^-5^ μg	0-5 Postnatal	Cervicovaginal lesions12%, 65% no CL	HANs 10%
		5 × 10^-4^ μg	0-5 Postnatal	19%, 88% no CL	HANs 19%
		5 × 10^-3^ μg	0-5 Postnatal	42%, 95% no CL	HANs 41%
		5 × 10^-2^ μg	0-5 Postnatal	63%, 100% no CL	HANs 7%
		5 × 1 μg	0-5 Postnatal	80%, 100% no CL	HANs 12%
Vassilacopoulou and Boylan 1993 [15]	ACI rat	4 + 4 μg	15 + 18 G	Not studied	Hypodifferentiation and hyperproliferation
**Effect on mammary tumorigenesis**					
Boylan and Calhoon 1979 [16]	Rat/DMBA	1.2 μg	Week 2 G	Not studied	Multiplicity↑
		1.2 μg	Week 3 G		Multiplicity↑
Boylan and Calhoon 1983 [18]	Rat/DMBA	0.6 + 0.6 μg	15 + 18 G	Not studied	Incidence + multiplicity↑
Rothschild *et al.* 1987 [19]	ACI rat	0.4 + 0.4 μg	15 + 18 G	Not studied	No change
		4 + 4 μg	15 + 18 G		Incidence↑
Ninomiya *et al.* 2007 [20]	Rat/DMBA	0.1 μg	Birth (one dose)	Normal cycle, 40% CL	Multiplicity↑
		1 μg	Birth (one dose)	19% PE, 50% CL, U w↓	Incidence + multiplicity↑
		10 μg	Birth (one dose)	77% PE, 92% CL, U w↓	Multiplicity↑
		100 μg	Birth (one dose)	100% PE, 100% CL, O + U w↓	No change
Yoshikawa *et al.* 2008 [22]	Rat/DMBA	14 x 1 μg	0-14 Postnatal	100% PE, no CL, O + U w↓, E2 and P↓	Incidence↓
		5 x 1 μg	0-5	PE, no CL, O + U w↓, E2 and P↓	Incidence↓
		9 x 1 μg	6-14	PE, no CL, O + U w↓	No change
Kawaguchi *et al.* 2009 [21]	Rat/DMBA	0.1 ppm	0-21 G	Few surviving pups	
		1 ppm	0-21 G	No surviving pups	
		10 ppm	0-21 G	No surviving pups	(Assessed 10 weeks after
		100 ppm	0-21 G	No surviving pups	DMBA exposure):
		0.1 ppm	13-21 G	11% no CL	Incidence + multiplicity↑
		1 ppm	13-21 G	30% no CL	Incidence + multiplicity↑
		10 ppm	13-21 G	Very few surviving pups	Incidence↑
		100 ppm	13-21 G	No surviving pups	

^a^In control mice, corpora lutea (CL) is present in about 31 to 36% of adults, whilst in control rats it is present in 100% of adult animals. DES, diethylstilbestrol; DMBA, dimethylbenz[a]antracene; E2, estradiol; F^1^, F1 generation; G, gestation; HAN, hyperplastic alveolar nodule; O, ovary; P, progesterone; PE, persistent estrus; U, uterus; w, weight.

The animal studies show that the doses of DES relevant to pregnant women increased later risk of developing mammary tumors. Specifically, female offspring of rat dams exposed to a total of 1.2 μg DES either on gestation week 2 or 3 [16], to 0.6 μg or 4 μg DES on both gestation days 15 and 18 (all via injection) [18,19], or via diet to 0.1, 1 or 10 ppm DES between gestation days 13 and 21 (week 3) [21] exhibited increased mammary cancer risk. An increase in risk also was seen in rats exposed to a single dose of 0.1, 1 or 10 μg or less of DES at birth [20]. In mice, the effect of neonatal exposure to DES on mammary gland development has been studied by many investigators (see below), but not on mammary tumorigenesis. It is apparent from the rat studies that high maternal DES doses, especially if they started early in pregnancy, prevented implantation or caused miscarriage [20,21], as is the case in humans [9]. Further, a postnatal exposure for several consecutive days reduced mammary tumorigenesis [22], consistent with the data showing that postnatal exposure (prior to puberty onset) to estrogens protects against mammary cancer [23].

#### *Effects on mammary glands in animals*

In addition to an increase in mammary tumorigenesis in animal models, high *in utero* estrogenic environment impacts the development of the mammary gland, which begins with the formation of the mammary lines on gestational day 10 in the mouse and rat [24]. Ductal branching morphogenesis begins on gestational day 16 when epithelial cells from the mammary bud grow down into the mesenchyme and the mammary fat pad. However, the growth of the mammary tree remains rather quiescent until the animal reaches puberty during postnatal week 4 [25,26]. At puberty, terminal end buds (TEBs), which are located at the tips of growing epithelial ducts, lead to the growth of the mammary epithelial tree. As the epithelium grows, bifurcation of TEBs gives rise to ducts and alveolar buds, which further differentiate to lobules. When TEBs reach the edges of the fat pad in 8- to 10-week-old mice and rats, they regress to terminal buds [27]. TEBs give rise to malignant mammary tumors in carcinogen-treated animals [27], and corresponding structures in the human breast (terminal ductal lobular unit) appear to be the site of breast cancer initiation in most women [28]. Further, the number of TEBs correlates with breast cancer susceptibility [27,29]. After TEBs regress in adult glands, the glands are no longer susceptible to mammary cancer initiation by carcinogens, such as chemicals or radiation [30,31]. Importantly, *in utero* exposure to DES leads to an increase in TEB numbers [20,32]. It is thus possible that one of the mechanisms causing an increase in mammary cancer risk in DES offspring is an increase in the number of targets for malignant transformation.

### Findings in human studies

Several published studies have investigated breast cancer risk in the daughters of DES mothers, the majority of which were cohort studies done in the US. As the women in the cohorts aged, their breast cancer risk grew higher, compared with matched non-exposed controls [9,33-36]. The findings clearly indicate that after age 40 years the incidence of breast cancer is at least two-fold higher in the daughters of DES-exposed mothers. Many pregnant women in Europe and Australia also used DES, but the peak exposure occurred 10 to 20 years later than in the US, and this probably explains why a recent study done in Europe found a trend but not a significant increase in breast cancer risk among them [37]. Once the European daughters reach the age when breast cancer is more commonly detected, they too are likely to exhibit a significant increase in breast cancer risk.

Density of TEBs in rodents might be modeling mammographic density in women. Mammographic density is determined as a ratio between epithelial area containing the epithelial and stromal/connective tissue cells, and the whole breast (epithelial area plus adipose cells). Mammographic density thus reflects the number of epithelial cells, especially terminal ductal lobular units [38], and it is strongly linked to increased breast cancer risk [39]. No human studies have been published about *in utero* DES exposure and mammographic density. However, since a surrogate marker of having been exposed to elevated pregnancy hormonal environment - high birth weight - is associated with increased mammographic density [40], it is possible that DES daughters also have increased mammographic density.

To summarize, animal and human studies have generated similar findings and indicate that there is a causal link between maternal exposure to DES during pregnancy and increased breast cancer risk among female offspring. According to animal studies, the increase in risk may reflect the presence of a higher number of TEBs in the mammary epithelium in the DES offspring. Baik and colleagues [41] have proposed that the increase in mammary epithelial cells in *in utero* estrogen-exposed females is caused by a high number of mammary stem cells or an increase in their potential to generate daughter cells. Our unpublished data support this conclusion and show that *in utero* exposure to the synthetic estrogen ethinyl estradiol increases the stem-cell like population in the developing mammary gland.

## Mechanisms mediating the effects of in utero diethylstilbestrol exposure on the mammary gland

When determining how maternal exposure to DES during pregnancy can impact mammary gland development and breast cancer risk, several questions need to be answered. First, what are the changes in the fetal mammary gland caused by DES? Second, how are these changes maintained to adult life? The second point is especially important, since there is no evidence that *in utero* DES exposure would induce mutations in the mammary tissue [42], or that DES daughters develop breast cancer at an earlier age than non-exposed daughters [9,35].

Several signaling factors, expressed in the epithelial cells and mesenchyma, have been identified that are critical for fetal mammary gland development [25,26]. Interestingly, although the estrogen receptor (ER) is expressed in the fetal mammary gland [26,43], findings obtained in ER knockout mice show that the fetal mammary gland can develop normally without this receptor [44,45]. This is surprising since *in utero* or neonatal exposure to estrogens, including DES, alters fetal and neonatal mammary gland development in humans [46] and mice [47]. A likely explanation for these findings is that although ER is not required for fetal mammary gland development, estrogens can modify the developmental process by regulating or interacting with the signaling factors that are essential for its development, such as the Wnt/β-catenin pathway, parathyroid hormone-related protein, bone morphogenic protein 4 and the insulin-like growth factor family [48-50].

Relatively little is known about long-term changes in the transcriptome of the mammary glands in animals exposed to estrogenic compounds *in utero.* Umekita and colleagues [32] investigated the effect of neonatal exposure to DES on gene expression in the TEBs, using a dose (1 μg/kg) that is known to increase the number of these structures [20]. The most significant changes in gene expression involved the NF-kB signaling pathways at puberty onset and ERK pathways in adult mammary glands [32]. NF-kB is linked to breast cancer progression [51] and anti-estrogen resistance [52], and among many functions of ERK are those leading to increased breast cancer risk and impaired response to anti-estrogens as well as poor prognosis [53].

There is general agreement that the changes in the transcriptome of adult tissues found to be affected by alterations in the *in utero* environment are likely to have been epigenetically induced. This is because the epigenetic signature in all fetal cells, including germ cells, is established during early development and the signature then interprets the information in the genetic code that is inherited from both parents by means that do not involve a change in DNA sequence [54].

### Epigenetic processes mediating the effects of in utero diethylstilbestrol exposure

Gene expression can be altered as a consequence of mutations or epigenetic changes. In contrast to gene mutations within the DNA, epigenetic changes involve post-transcriptional modifications; that is, methylation of gene promoter regions, histone modifications, deposition of certain histone variants along specific gene sequences and microRNA (miRNA) expression. Although both changes are heritable, an important distinction between the two is that mutations are not reversible, but epigenetic modifications generally are.

Probably the most common mechanism of epigenetic gene silencing is methylation [55], and it might also be the most important. DNA methyltransferases (DNMTs) catalyze the methylation of genomic DNA by adding a methyl group (CH_3_) onto the 5-carbon of the cytosine ring within CpG dinucleotides. Histone modifications are complex, as they involve not just histone methylation but also acetylation, deacetylation and other post-translational changes. These modifications occur in the amino-terminal tails of histones and affect the 'openness' of the chromatin, which determines whether a gene is expressed or silenced (for example, acetylation allows transcription, while deacetylation represses transcription) [56,57]. Trimethylation of histone H3 at lysine K27 is catalyzed by the Polycomb group (PcG) protein enhancer of Zeste-2 (EZH2) and results in gene silencing [58,59]. PcG/H3K27me3 interact with DNMTs [60,61], and together they establish and maintain silencing of PcG target genes [62]. Over 2,000 different PcG target genes have been identified [63] and they include some tumor suppressor genes. Many of the PcG target genes regulate cell fate, including apoptosis, proliferation and stem cell differentiation [64-66]. As discussed in more detail below, methylation of PcG target genes is linked to increased breast cancer risk.

DNMTs may be key players in regulating histones and the entire epigenomic machinery, since DNA methylation events often precede histone modifications [67]. Upregulation of DNMTs increases the expression of EZH2 and other polycombs; this may happen by DNMTs inducing methylation of non-coding miRNAs that target the polycombs [68].

### Epigenetic alterations induced by in utero diethylstilbestrol exposure

We and others have observed that the expression of DNMTs is persistently altered in estrogen-regulated tissues following estrogenic exposures during early life. *In utero* exposure to DES is reported to increase the expression of DNMT1 in the epididymis [69] and uterus [70]. We found that DNMT1 expression is increased in the mammary glands of adult rat offspring of dams exposed to ethinyl estradiol during pregnancy [71]. These changes provide a key regulatory layer to influence gene expression in the mammary gland and perhaps breast tumors of individuals exposed to DES or other estrogenic compounds *in utero*.

#### *Promoter methylation*

*In utero* DES exposure alters methylation patterns of several genes in estrogen’s target tissues, including Hox genes [72,73], c-fox [74], and Nsbp1 [75], but it has not been studied whether changes in methylation patterns occur in the mammary gland. We have explored changes in methylation in the mammary glands of adult rats exposed *in utero* to the synthetic estrogen ethinyl estradiol using global sequencing approaches [71]. Among the genes that exhibited increased promoter methylation were several PcG target genes, suggesting that a maternal exposure to synthetic estrogens during pregnancy causes long-lasting changes in the methylation of genes that regulate cell fate, including stem cell differentiation.

#### *Histone modifications*

As an increase in EZH2 expression in the mammary glands of mice exposed to DES *in utero* has been reported [76], histone modifications also seem to be influenced by maternal exposure to synthetic estrogens during pregnancy. Jefferson and colleagues [77] recently investigated whether upregulation of lactoferrin and sine oculis homeobox 1 (Six1) in the uterus of adult mice exposed to DES neonatally is caused by histone modifications. Their data indicate that neonatal DES exposure induces changes during the early postnatal period in the expression of multiple chromatin-modifying proteins but these changes do not last to adulthood. However, alterations in epigenetic marks at the Six1 locus in the uterus were persistent [77]. Similarly, changes in the methylation of Nsbp1 [75] and expression of DNMTs [70] in the uterus of DES-exposed offspring are different in the early postnatal period compared to adulthood. This suggests that some epigenetic alterations are further influenced by factors operating during postnatal development, such as a surge of estrogens and progesterone from the ovaries at puberty onset.

#### *microRNAs*

Maternal exposures during pregnancy have been found to induce persistent changes in miRNA expression in the offspring. miRNAs are short non-coding single-stranded RNAs composed of approximately 21 to 22 nucleotides that regulate gene expression by sequence-specific base-pairing with the 3’ untranslated region of target mRNAs. miRNA binding induces post-transcriptional repression of target genes [78], either by inducing inhibition of protein translation or by inducing mRNA degradation. Expression of many miRNAs is suppressed by estrogens [79,80]. Although the effects of maternal DES exposure during pregnancy on miRNA expression in the offspring have not been investigated, it is known that many other manipulations, such as maternal low protein diet, alter miRNA patterns among the offspring [81]. We recently found that *in utero* exposure to ethinyl estradiol lowers the expression of many of the same miRNAs in the adult mammary gland [82] as are downregulated by E2 in MCF-7 human breast cancer cells [79]. Since miRNAs can be silenced by methylation [83,84] or as a result of increased PcG expression [85], and they target DNMTs, histone deacetylases and polycomb genes [86,87], the observed increase in DNMT expression, histone marks and EZH2 in the *in utero* DES-exposed offspring may be a result of epigenetic silencing of miRNAs that target them.

### Epigenetic alterations and breast cancer risk

Methylation of PcG target genes and tumor suppressor genes in peripheral blood cells, detected years before diagnosis, is associated with increased breast cancer risk, particularly in women with a high familial risk [88-90]. Similar changes have been detected in cells collected by random periareolar fine-needle aspiration from asymptomatic women at high risk for breast cancer [90]. Among the tumor suppressor genes found to be hypermethylated in women at high risk, but who are negative for germline *BRCA1* or *BRCA2* mutations, are those encoding RARB, ER-α, INK4a/ARF, BRCA1, PRA, PRB, RASSF1A, HIN-1, and CRBP1. Many of these genes also are PcG target genes. Methylation of many other, 'non-tumor suppressor' PcG target genes also are observed in cancer [91,92]. It has been estimated that 53% of the genes hypermethylated in early-stage breast cancer are known PcG target genes [93]. Since *in utero* exposure to synthetic estrogen causes hypermethylation of PcG target genes [71] and increases mammary cancer risk [94], in some women at high risk for breast cancer methylation of these genes may originate from having been exposed to synthetic estrogens during the fetal period.

Several miRNAs are altered in breast tumors in women, compared with normal tissue [95]. There is some evidence that changes in the miRNA profile in the peripheral blood may serve as biomarkers for the presence of breast cancer [96], and that these changes in healthy women may predict for an increase in breast cancer risk [97]. It remains to be determined whether epigenetic changes, including downregulation of miRNAs seen in some women at high risk for breast cancer, are induced by an exposure to an excessive *in utero* estrogenic environment.

## The effects of maternal diethylstilbestrol exposure are not limited to the F1 generation?

Developing germ cells undergo epigenetic erasure when they, as primordial germ cells, enter into the fetal gonads around embryonic day 10 to 11 (in mice and rats), and then undergo gender-specific reprogramming as germ cells [98]. It is now clear that reprogramming of these cells is susceptible to modifications caused by changes in fetal hormonal environment, such as resulting from an exposure to DES or other endocrine disruptors. Consequently, these exposures can leave a permanent biochemical footprint on the genome of the F1 generation germ cells, and this change may be inherited by the F2 generation germ line and several subsequent generations.

Skinner’s group [99] has investigated differences in methylation patterns in the germ cells of adult male F3 generation offspring of dams exposed during pregnancy to vinclozolin, a fungicide with anti-androgenic properties. Their data indicated that 52 promoter regions were differentially methylated (either hypo- or hypermethylated), compared with controls [99]. The effects of maternal vinclozolin on germ cells in F3 generation adult female offspring also have been explored, and 43 differentially methylated genes were identified [100]. Another study investigated the effect of maternal exposure to dioxin during pregnancy on methylation changes in the germ cells of adult male F3 generation offspring, and also identified several differentially methylated genes [101]. In the most recent study, Skinner and colleagues [102] showed that maternal exposure to vinclozolin during pregnancy altered methylation patterns and gene expression of primordial germ cells in F3 generation male fetuses, compared with the F3 generation control offspring.

We have investigated changes in the methylation patterns of the mammary glands in three generations of female offspring of rat dams exposed to ethinyl estradiol or vehicle during pregnancy [71]. A total of 351 genes were identified that had their promoter region either hyper- or hypomethylated in the ethinyl estradiol offspring, compared with the controls. Mammary tumorigenesis and the number of TEBs also were increased in daughters, granddaughters and great granddaughters of dams exposed to ethinyl estradiol during pregnancy [71].

In addition to changes in histones and DNA methylation, miRNA expression may be affected in germ cells by hormonal exposures during early development. Meunier and colleagues [103] investigated the effects of neonatal exposure to synthetic estrogen on miRNA expression in the adult male germ cells, and discovered that miR-29 was upregulated and its target genes *DNMT1*, *DNMT3A* and *DNMT3B* were all downregulated. Effects on female germ cells were not studied, but we and others have found increased DNMT expression in the breast and reproductive tissues in *in utero* ethinyl estradiol- or DES-exposed animals [69-71]. It is possible that male and female epigenomes respond differently to a synthetic estrogen exposure *in utero*.

Some researchers have begun to investigate whether the effects of maternal DES exposure during pregnancy extend to the third generation in humans. Although there is no evidence that DES granddaughters have cervical and ovarian abnormalities similar to DES daughters, there is evidence that they may have more menstrual irregularities and a higher rate of infertility than non-exposed granddaughters [104]. In addition, DES granddaughters may have a slightly higher risk of ovarian cancer [105]. The granddaughters are still too young to assess whether they might also be at an increased risk of developing breast cancer.

## Future directions

Millions of women in the US, Europe and Australia have been exposed to DES in the womb, and consequently exhibit about a two times higher breast cancer risk than unexposed women. The increase in risk may not be limited to the DES-exposed daughters, but could also increase breast cancer risk in granddaughters and great granddaughters. Such outcome would be consistent with the findings we obtained in studies using a synthetic estrogen ethinyl estradiol (EE2) [71]. If DES has similar effects to ethinyl estradiol on the transgenerational increase in breast cancer risk, it is urgent to find ways to stop the cycle of inheritance, and also prevent breast cancer in DES-exposed granddaughters and great granddaughters.

**Figure 1:**
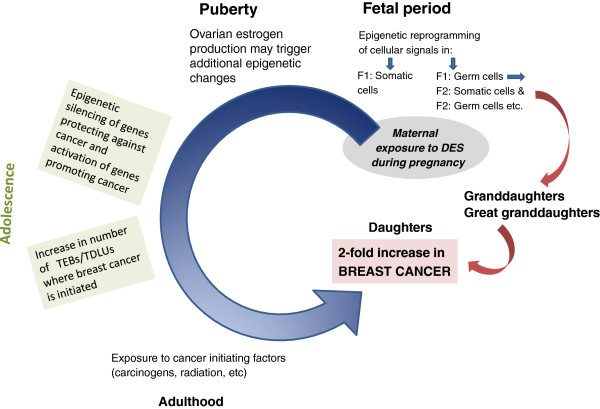
**Proposed model to explain an increase in breast cancer risk in daughters, and possibly granddaughters and great granddaughters, of mothers who took diethylstilbestrol during pregnancy.** DES, diethylstilbestrol; TDLU, terminal ductal lobular unit; TEB, terminal end bud.

To achieve this goal, we need to understand how maternal DES exposure during pregnancy increases a daughter’s breast cancer risk. A plausible model is proposed in Figure 1. It is evident from studies done in animal models that *in utero* DES exposure induces epigenetic changes in reproductive tract tissues [69,70,72-75,77] and the breast [76]. DES exposure might also have induced epigenetic changes in primordial germ cells and consequently germ cells, and further be detectable in the somatic cells in granddaughters and great granddaughters. We are not aware of any study that has compared epigenetic changes in germ cells and the next generation somatic cells in individuals exposed to DES or other endocrine disruptors *in utero*. Second, we should investigate whether the transgenerational increase in breast cancer risk can be prevented with drugs that reverse epigenetic modifications. Our preliminary studies in mice suggest that this is achievable in daughters by using the well-tolerated and non-toxic histone deacetylase inhibitor valproic acid and DNMT inhibitor hydralazine. However, whether these compounds also prevent an increase in granddaughters and great granddaughters in experimental models remains to be investigated.

## Conclusions

In summary, women exposed to DES *in utero* are destined to be at an increased risk of developing breast cancer, and this risk may extend to their daughters and granddaughters as well. It is of critical importance to determine if the increased risk is driven by epigenetic alterations in genes that increase susceptibility to breast cancer and if these alterations are reversible.

## Abbreviations

DES: Diethylstilbestrol; DNMT: DNA methyltransferase; ER: Estrogen receptor; EZH2: Protein enhancer of Zeste-2; miRNA: microRNA; NF: Nuclear factor; PcG: Polycomb group; Six1: Sine oculis homeobox 1; TEB: Terminal end bud.

## Competing interests

LH-C has served as an expert witness in a case concerning daughters of DES-exposed mothers on behalf of the plaintiffs.
